# Inflammatory cytokines are associated to lower glomerular filtration rate in patients with hypertensive crisis

**DOI:** 10.3389/fcvm.2022.969339

**Published:** 2022-09-29

**Authors:** Days O. Andrade, Franciana L. Aguiar, Ana Luiza P. Mansor, Flavia M. Valente, Doroteia R. S. Souza, Valquiria da Silva Lopes, Leticia B. Fernandes, Moacir F. Godoy, Juan C. Yugar-Toledo, Luciana N. Cosenso-Martin, Jose F. Vilela-Martin

**Affiliations:** ^1^Hypertension Clinical and Medicine Department, State Medical School at São José do Rio Preto, São Paulo, Brazil; ^2^Biochemistry and Molecular Biology Research Nucleus and Molecular Biology Department, State Medical School at São José do Rio Preto, São Paulo, Brazil; ^3^Transdisciplinary Nucleus for the Study of Chaos and Complexity, de Cardiology and Cardiovascular Surgery Department, State Medical School at São José do Rio Preto, São Paulo, Brazil

**Keywords:** blood presssure, hypertension, hypertensive crisis, inflammation, cytokines, renal function

## Abstract

**Introduction:**

Hypertension and kidney function are closely related. However, there are few studies on renal function during acute elevation of blood pressure (BP), denominated hypertensive crisis (HC).

**Objectives:**

To evaluate the relationship between renal function and inflammatory cytokines in HC, subdivided into hypertensive urgency (HUrg) and emergency (HEmerg).

**Materials and methods:**

This cross-sectional study was carried out in 74 normotensive (NT) and 74 controlled hypertensive individuals (ContrHT) followed up in outpatient care. Additionally, 78 subjects with hypertensive emergency (HEmerg) and 50 in hypertensive urgency (HUrg), attended in emergency room, were also evaluated. Hypertensive crisis was classified into HEmerg, defined by systolic blood pressure (BP) ≥ 180 mmHg and/or diastolic BP ≥ 120 mmHg in presence of target-organ damage (TOD), and HypUrg, clinical situation with BP elevation without TOD. The glomerular filtration rate (eGFR) was estimated, and cytokine levels were measured. Statistical analysis was performed using the Kruskal-Wallis or Mann-Whitney test and Spearman’s correlation, with significant differences *p*-value < 0.05.

**Results:**

The median age was 53.5 years in the NT group (52 female), 61 years in the ContrHT group (52 female), and 62.5 years in the HC group (63 female) (*p*-value < 0.0001). The median BP was 118.5/75 mmHg for NT, 113.5/71 for ContrHT, and 198.5/120 mmHg for HC, respectively (*p*-value < 0.0001 among groups). BP and heart rate levels were significantly higher in the HC group compared to the NT and ContrHT groups (*P* < 0.001 for all). The eGFR was significantly lower in HC group compared to the NT and ContrHT groups. The cytokine levels were higher in the HEmerg and HUrg groups compared to ContrHT group (*P* < 0.0001, except for IL-1β in HUrg vs. ContrHT), without difference between the acute elevation of BP groups. Thus, all cytokines were significantly elevated in patients with HC compared to the control groups (NT and ContrHT). There was a negative correlation between eGFR and the cytokines (IL-1β, IL-6, IL-8, IL-10, and TNF-α) in the HC group.

**Conclusion:**

Elevated inflammatory cytokines are associated with reduced eGFR in individuals with HC compared to control groups, suggesting that the inflammatory process participates in the pathogenesis of acute elevations of BP.

## Introduction

Hypertension is a growing world public health problem. High blood pressure may cause severe complications including coronary artery disease, heart failure, stroke, kidney failure, and death unless it is diagnosed and treated early (1). One acute consequence of inadequate blood pressure (BP) control is hypertensive crisis (HC), a serious clinical presentation in which an abrupt elevation in BP (systolic BP ≥ 180 mmHg and/or diastolic BP ≥ 120 mmHg) can lead to acute lesions in target organs (brain, heart, kidneys, and arteries) promoting prompt or potential risk of death (2). Hypertensive crisis is divided in an emergency (HEmerg), characterized by target organ damage (TOD) and risk of death or hypertensive urgency (HUrg), a situation without TOD and, consequently, no risk of potential death (3). It is estimated that 1–2% of hypertensive individuals will have HC at some point in life (4).

The precise pathophysiology of the HC is poorly understood. Nevertheless, different interconnected mechanisms may play central roles in its pathophysiologic process. The first is a failure in the autoregulation system of the arterial bed, which courses with a diminution of the perfusion pressure leading to a reduction in blood flow and an increment in arterial resistance, originating mechanical stress and endothelial lesion (5). The second is the renin–angiotensin system activation, causing vasoconstriction and leading to a vicious cycle of endothelial damage and thereafter ischemia. A prothrombotic state may also play a key function in HC pathophysiology (4, 5).

On the other hand, the kidneys play an essential role in the regulation of BP with renal function acting a predictor factor of cardiovascular risk in high blood pressure. Chronic kidney disease and cardiovascular abnormality may be causes and also effects of sustained hypertension, resulting in a high risk of morbidity and mortality (6). Considering that the renal system has an important participation in regulation of BP, any type of renal artery disease or renal parenchymal disorder may cause HC (7). Furthermore, acute kidney injury (AKI) may be a complication of hypertensive crisis (7, 8). In cases of HEmerg with AKI, the presence of subjacent kidney disease at baseline and the phase of kidney dysfunction at admission affect the rate of renal recovery after improvement of the BP (7, 9). In addition, inflammatory cytokines participate in the pathogenesis of chronic hypertension by actions on kidney blood flow and sodium balance (10, 11). Thus, there are few studies of the association between kidney function and the inflammatory process in cases of acute increment of BP defined as HC. Therefore, this study will evaluate if inflammatory markers are associated to renal function in individuals suffering from acute elevations of BP.

## Materials and methods

This was a cross-sectional study conducted in patients treated in a tertiary referral university hospital in the period from 2012 to 2014. This study was submitted to and approved by the Research Ethics Committee of Medical School at Sao Jose Rio Preto (FAMERP) according to national and international guidelines (number 3167/2005–05/16/2012). All participants signed informed consent forms. All methods were performed in accordance with relevant guidelines and regulations. A total of 274 individuals aged 18 years and over were divided into four groups: Normotensive (NT; *n* = 74); Controlled Hypertensive (ContrHT; *n* = 74); Hypertensive Urgency (HUrg; *n* = 50); Hypertensive Emergency (HEmerg; *n* = 78). The control groups consisted of NT subjects selected in specialized outpatient clinics with BP < 140 × 90 mmHg in the office without previous use of antihypertensive drugs and by controlled hypertensive individuals (ContrHT) being followed-up in the hypertension outpatient clinic with BP < 130 × 80 mmHg measured by 24-h ambulatory monitoring of blood pressure. The individuals with HC were recruited at the emergency room.

The criterium proposed by the Seventh Joint National Committee was used for the definition of HC (2). HEmerg was characterized by severe increases in BP complicated by evidence of impending or progressive TOD (hypertensive encephalopathy, hemorrhagic and ischemic strokes, acute myocardial infarction, acute pulmonary edema, unstable angina, acute aortic dissection, and acute/progressive renal insufficiency). HUrg were situations associated with severe elevations in BP without TOD. The clinical history of all patients was taken, and they were submitted to physical examinations and diagnostic exams after signing an informed consent form previously approved by the Ethics Research Committee. In cases where patients were unable to sign, participation was authorized by parents or guardians.

To evaluate TOD better, patients in the HEmerg group were subdivided into two types of organic involvement: cerebrovascular (ischemic stroke, hemorrhagic stroke, and hypertensive encephalopathy) and cardiovascular (acute pulmonary edema, acute myocardial infarction, unstable angina, and acute aortic dissection).

The BP of patients was measured according to the recommended standard technique using a digital automatic sphygmomanometer (Omron Hem-711 DLX); the mean was calculated from at least three consecutive readings taken at 1-min intervals.

This study excluded female patients presenting with preeclampsia and eclampsia, and hypertensive patients with pseudocrises or chronic inflammatory diseases (rheumatoid arthritis, lupus, fibromyalgia, cancer, and others). The clinical profile of the patients was obtained from electronic medical records.

### Laboratory analyses

In order to measure inflammatory mediator levels, peripheral blood samples were collected in dry tubes and centrifuged at 3,500 r.p.m. for 10° min. The plasma was stored in freezer tubes at −70°C. In the cases of the HEmerg and HUrg, blood samples were collected within 4° h of emergency hospital admission. Interleukins (IL-1β, IL-6, IL-8, and IL-10) and tumor necrosis factor-alpha (TNF-α) were measured using MILLIPLEX^®^ MAP and the interleukin 18 (IL-18) was measured using the enzyme-linked immunosorbent assay (ELISA). These specific cytokines for the present study were selected according to literature analyzes (11, 12). All cytokines were assayed in duplicate. Analyses of serum creatinine and potassium were performed using standard assays (12). The estimated glomerular filtration rate (eGFR) was calculated using CKD-EPI equation, developed by the Chronic Kidney Disease Epidemiology Collaboration (13).

### Statistical analysis

Considering the present study with 276 cases included, with an alpha error of 5% and effect size of 0.20 for IL-1β, the power of the study was 84%. This sample size was able to identify significant differences in interleukins values between the study groups.

The descriptive analyses of the variables are presented as median, minimum, and maximum values. The chi-square and Fisher’s exact tests were used in the comparative study to analyze the epidemiological profile and use of medications and metabolic factors and renal function.

For continuous variables, such as metabolic factors and renal function, the Kruskal Wallis test was applied; in the case of rejection of the null hypothesis, multiple comparisons were carried out with significance values adjusted by Bonferroni correction, to find the effect of differences.

The eGFR, systolic BP (SBP), diastolic BP (DBP), creatinine, and inflammatory cytokines were included in the Spearman’s correlation analysis. Spearman’s correlation was also performed to verify the correlation between age and inflammatory cytokines. The results were demonstrated by the Spearman’s correlation coefficient and its respective 95% confidence interval. Outliers creatinine levels were identified and excluded using the GraphPad Prism. An alpha error of 5% was accepted; thus, statistical differences were considered significant when *p*-values were < 0.05. Statistical analysis was performed using the IBM-SPSS Statistics software version 28 (IBM Corporation, Yorktown Heights, NY, USA).

## Results

The socio-demographic data and biochemical-metabolic parameters of the four study groups are shown in Table 1. As observed, there was no difference of the socio-demographic data and biochemical-metabolic profile between the HUrg and HEmerg groups. Thus, we decided joint both groups (HC group) for presenting this data (Table 2). BP and heart rate levels were significantly higher in the HC group compared to the NT and ContrHT groups (*P* < 0.001 for all). Moreover, the HC group showed statistically higher levels of creatinine than the NT and ContrHT groups (*P* < 0.001 for all). The eGFR was significantly lower in the HC group compared to the NT and ContrHT groups (*P* < 0.0001, 0.005, respectively).

**TABLE 1:** Sociodemographic data and biochemical profile of normotensive (NT), controlled hypertensive (ContrHT), hypertensive urgency (HUrg), and hypertensive emergency (HEmerg) subjects.

Variable	NT (a) (*n* = 74)		ContrHT (b) (*n* = 74)		HUrg (c) (*n* = 50)>		HEmerg (d) (*n* = 78)							
**Gender**	**N (%)**		**N (%)**		**N (%)**		**N (%)**							
Female	50 (71)		52 (70)		23 (46)		38 (49)							
**Ethnical background**									**a x b**	**a x c**	**a x d**	**b x c**	**b x d**	**c x d**
White	69 (93.2)		63 (85.1)		39 (78)		65 (83.3)							
Non-white	5 (6.8)		11 (14.9)		11 (12)		13 (16.7)							

	**Median**	**Range**	**Median**	**Range**	**Median**	**Range**	**Median**	**Range**						

Age (years)	53.5	37–83	61	24–84	58	32–92	63	25–97	**0.011**	0.325	**<0.001**	1.000	1.000	0.202
BMI (kg/m^2^)	26.8	18.9–36.9	29.7	21–47.9	29.3	19.4–47.4	26	17.5–46.8	**0.003**	0.206	1.000	1.000	**0.006**	0.339
SBP (mmHg)	118.5	95–133	113.5	93–130	200	160–300	196	124–300	1.000	**<0.001**	**<0.001**	**<0.001**	**<0.001**	1.000
DBP (mmHg)	75	55–94	71	50–88	120	110–180	120	110–240	0.371	**<0.001**	**<0.001**	**<0.001**	**<0.001**	1.000
HR (bpm/min)	68	55–90	71	42–108	81	55–150	87	53–141	1.000	**<0.001**	**<0.001**	**<0.001**	**<0.001**	1.000
**Biochemistry**														
Glycemia (mg/dL)	91	49–252	98	68–376	109	69–239	113	65–429	**0.0016**	**<0.0001**	**<0.0001**	NS	**0.033**	NS
HbA1c (%)	5.8	4.5–10	6.1	4.8–14.5	6.0	4.5–11.9	6.1	4.8–12.1	**0.0177**	NS	**0.0056**	NS	NS	NS
HDLc (mg/dL)	50	30–92	47	21–105	48.5	28–142	44	12–92	NS	NS	**0.0088**	NS	NS	NS
LDLc (mg/dL)	108	27–178	105	45–183	123.5	65–189	107	35–215	NS	NS	NS	NS	NS	NS
TG (mg/dL)	113.5	21–371	124.5	43–329	123	48–377	113	40–353	NS	NS	NS	NS	NS	NS
Creatinine (mg/dL)	0.8	0.5–1.3	0.9	0.6–1.7	1.0	0.6–10.3	1.0	0.5–8.1	NS	**0.003**	**<0.001**	NS	**0.001**	NS
Potassium (meq/L)	4.5	3.0–5.3	4.4	3.4–5.8	4.4	0.9–7.7	4.2	2.9–8.0	NS	NS	**0.034**	NS	NS	NS
**eGFR(mL/min/1.73m^2^**														
CKD-EPI)	88.0	48–128	82.0	35–129	75.0	5–123	66.0	7–125	0,308	**0,014**	**<0.001**	1.000	**0.002**	0.385

Values are presented as median, minimum, and maximum. *P*-value: level of significance <0.05; Kruskal–Wallis and Mann–Whitney tests; The bold values mean that the *P*-value is significant.

HbA1c, glycated hemoglobin; TG, triglycerides; HDLc, high-density lipoprotein cholesterol; LDLc, low-density lipoprotein cholesterol; eGFR, estimated glomerular filtration rate; NS, non-significant.

**TABLE 2:** Sociodemographic data, metabolic profile, and personal history of normotensive (NT), controlled hypertensive (ContrHT), hypertensive crisis (HC) individuals.

	NT (a) (*N* = 74)		ContrHT (b) (*N* = 74)		HC (c) (*N* = 128)		Kruskal-Wallis test		*P*-value*	
**Gender**	**N(%)**		**N(%)**		**N(%)**					
Female	52 (70.3)		52 (70.3)		63 (49.2)					
**Ethnical background**							**a x b**		**a x c**	**b x c**
White	65 (92.9)		63 (85.1)		104 (81.2)					
Non-White	5 (7.1)		11 (14.9)		24 (18.8)					

	**Median**	**Range**	**Median**	**Range**	**Median**	**Range**	***P*-value**	**a x b**	**a x c**	**b x c**

Age (years)	53.5	37–83	61.0	25–84	62.5	25–97	**<0.001**	**0.006**	**<0.001**	1.000
BMI (kg/m^2^)	26.8	18.9–36.9	29.7	21.0–47.9	27.3	17.5–47.4	**0.001**	**0.001**	0.690	**0.018**
SBP (mmHg)	118.5	95–133	113.5	93–130	198.5	124–300	**<0.001**	1.000	**<0.001**	**<0.001**
DBP (mmHg)	75.0	55–94	71.0	50–88	120.0	110–240	**<0.001**	0.185	**<0.001**	**<0.001**
HR (bpm/min)	68.0	55–90	71.0	42–108	85.0	53–105	**<0.001**	0.927	**<0.001**	**<0.001**
**Biochemistry**										
Creatinine (mg/dL)	0.8	0.5–1.3	0.9	0.6–1.7	1.0	0.5–10.3	**<0.001**	0.434	**<0.001**	**0.001**
Potassium (meq/L)	4.5	3.0–5.3	4.4	3.4–5.8	4.3	0.9–8.0	0.080	-	-	-
**eGFR(mL/min/1.73 m^2^)**										
CKD-EPI)	88	48–128	82	35–129	68	5–125	**<0.001**	0.154	**<0.001**	**0.005**
**Personal history**			**n (%)**		**n (%)**		***p*-value**			
Known hypertension			74 (100)		121 (94.5)		0.0993			
Dyslipidemia			50 (67.5)		64 (50)		**0.0227**			
Diabetes mellitus			29 (39.2)		31 (24.2)		0.025*			

Kruskal-Wallis and *Pearson’s chi-square test; The bold values mean that the *P*-value is significant.

BMI, Body mass index; SBP, Systolic blood pressure; DBP, Diastolic blood pressure; HR, Heart rate; eGFR, estimated glomerular filtration rate; CKD-EPI, Chronic Kidney Disease Epidemiology Collaboration.

The medications and the number of antihypertensive drugs used in the ContrHT, HUrg and HEmerg groups are shown in Table 3, which also exhibits the percentage of cardiovascular (acute pulmonary edema, acute myocardial infarction, and unstable angina) and cerebrovascular events (ischemic stroke, hemorrhagic stroke, and hypertensive encephalopathy) in the HEmerg group. Normotensive patients did not use antihypertensive, antidiabetic, or antilipemic drugs.

**TABLE 3:** Distribution of controlled hypertensive individuals (ContrHT) and hypertensive crisis (HC) according to use of medications. The table also shows the percentage of target organ damage found in the hypertensive emergency cases.

Variable	ContrHT	HC	
	*N* = 72	*N* = 128	*P*-value
**Anti-hypertensive drugs**	**n (%)**	**n (%)**	
Diuretics	62 (86.1)	70 (54.7)	**<0.001**
β blockers	22 (30.6)	74 (57.8)	**<0.001**
CCB	23 (31.9)	42 (32.8)	0.900
RAS blockers (ARB and ACEI)	59 (41)	118 (46,1)	0.322
Others	7 (9.7)	40 (31.3)	**<0.001**
**Antilipemic**			
Statins	49 (69)	63 (49.2)	**0.007**
Fibrates	1 (1.4)	1 (0.8	0.587*
**Antidiabetic drugs**			
Oral antidiabetic drugs	33 (46.5)	15 (11.7)	**<0.001**
Insulin	6 (8.5)	5 (3.9)	0.154*
Antiplatelet drugs	26 (36.6)	72 (56.3)	**0.008**
Anticoagulant	1 (1.4)	11 (8.6)	0.034*
1 anti-hypertensive drug	8 (11.3)	9 (7.1)	**0.040**
2 anti-hypertensive drugs	29 (40.8)	38 (29.9)	
3 anti-hypertensive drugs	25 (35.2)	42 (33.1)	
> 3 anti-hypertensive drugs	9 (12.7)	38 (29,9)	
Target organ damage Acute pulmonary edema		24 (30.8)	
Ischemic stroke		24 (30.8)	
Hemorrhagic stroke		13 (16.6)	
AMI		6 (7.7)	
Unstable angina		6 (7.7)	
Hypertensive encephalopathy		5 (6.4)	

Pearson’s chi-square test and *Fisher exact test; The bold values mean that the *P*-value is significant.

ContrHT, Controlled hypertensive; HC, Hypertensive crisis; CCB, Calcium channel blocker; RAS, Renin-angiotensin system; ARB, Angiotensin receptor blocker; ACEI, Angiotensin converting enzyme inhibitor; AMI, Acute myocardial infarction.

**FIGURE 1:**
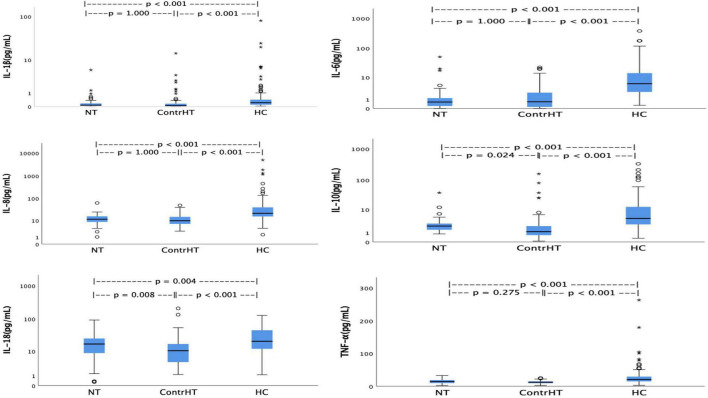
Values of interleukins distributed among the normotensive (NT), controlled hypertension (ContrHT), and hypertensive crisis (HC) groups.

The Table 4 presents the results of the inflammatory cytokines for the four study groups. All proinflammatory cytokines were significantly higher in patients with HEmerg, compared to the control groups (NT and ContrHT). The cytokine levels (IL-6, IL-8, IL-10, IL-18 e TNF-α) were higher in both HEmerg and HUrg groups compared to ContrHT group (*P* < 0.0001 for both). The cytokine levels were not significantly different between the HEmerg and HUrg groups, except for IL-1β. For statistical analysis, we decided to unite the two groups, as demonstrated in the Table 5. All proinflammatory cytokines were significantly higher in patients with HC, compared to the control groups (NT and ContrHT). The Figure 1, as boxplot graphic, evidences the interleukins values distributed among the NT, ContrHT, and HC groups.

**TABLE 4:** Measurement of inflammatory cytokine levels in normotensive (NT), controlled hypertensive (ContrHT), hypertensive urgency (HUrg), and hypertensive emergency (HEmerg) subjects.

Variable	NT (a) (*n* = 74)		ContrHT (b) (*n* = 74)		HUrg (c) (*n* = 50)		HEmerg (d) (*n* = 78)		*P*-value*					
	Median	Range	Median	Range	Median	Range	Median	Range	a x b	a x c	a x d	b x c	b x d	c x d
IL-1β(pg/mL)	0.07	0.00–5.54	0.05	0.00–14.4	0.10	0.02–24.8	0.30	0.14–81.3	1.000	0.375	**<0.0001**	0.107	**<0.0001**	**<0.0001**
IL-6 (pg/mL)	0.63	0.00–51.5	0.67	0.00–22.6	4.45	0.27–48.6	7.65	0.51–386	1.000	**<0.0001**	**<0.0001**	**<0.0001**	**<0.0001**	0.970
IL-8 (pg/mL)	11.4	1.07–63.7	9.7	2.7–49.9	21.39	1.61–1174	21.75	4.03–5034	1.000	**<0.0001**	**<0.0001**	**<0.0001**	**<0.0001**	1.000
IL-10 (pg/mL)	2.28	0.82–37.9	1.15	0.07–158	4.19	0.32–335	6.32	0.36–213	**0.047**	**0.003**	**<0.0001**	**<0.0001**	**<0.0001**	1.000
IL-18 (pg/mL)	16.95	0.3–93.7	10.3	1.17–210.7	22.05	4.1–129.5	20.17	1.13–80.5	**0.016**	0.053	**0.026**	**<0.0001**	**<0.0001**	1.000
TNF-α(pg/mL)	14.27	2.32–32.6	12.42	2.47–25.0	20.81	9.36–101	20.3	2.6–264	0.550	**<0.0001**	**<0.0001**	**<0.0001**	**<0.0001**	1.000

*P-value < 0.05; Kruskal–Wallis, Mann–Whitney tests; The bold values mean that the *P*-value is significant.

IL-1 β, Interleukin 1β; IL-6, Interleukin 6; IL-8, Interleukin 8; IL-10, Interleukin 10; IL-18, Interleucina 18; TNF-α, Tumor necrosis factor-α.

**TABLE 5:** Measurement of inflammatory cytokine levels in normotensive (NT), controlled hypertensive (ContrHT), and hypertensive crisis (HC) subjects.

	NT (a) (*N* = 74)		ContrHT (b) (*N* = 74)		HC (c) (*N* = 128)		Kruskal-Wallis test	*P*-value Multiple comparisons		
	Median	Range	Median	Range	Median	Range	*P*-value	a x b	a x c	b x c
IL-1β(pg/mL)	0.07	0.00; 5.54	0.05	0.00; 14.40	0.22	0.02; 81.35	**<0.001**	1.000	**<0.001**	**<0.001**
IL-6 (pg/mL)	0.63	0.00; 51.57	0.67	0.00; 22.66	5.70	0.27; 386.00	**<0.001**	1.000	**<0.001**	**<0.001**
IL-8 (pg/mL)	11.48	1.07; 63.73	9.77	2.72; 49.96	21.72	1.61; 5034.00	**<0.001**	1.000	**<0.001**	**<0.001**
IL-10 (pg/mL)	2.28	0.82; 37.91	1.15	0.07; 158.00	4.80	0.32; 335.00	**<0.001**	**0.024**	**<0.001**	**<0.001**
IL-18 (pg/mL)	16.95	0.30; 93.70	10.33	1.17; 210.77	20.70	1.13; 129.50	**<0.001**	**0.008**	**0.004**	**<0.001**
TNF-α(pg/mL)	14.27	2.32; 32.64	12.42	2.47; 25.04	20.66	2.69; 264.00	**<0.001**	0.275	**<0.001**	**<0.001**

The bold values mean that the *P*-value is significant.

CKD-EPI, Chronic Kidney Disease Epidemiology Collaboration; IL-1 β, Interleukin 1β; IL-6, Interleukin 6; IL-8, Interleukin 8; IL-10, Interleukin 10; IL-18, Interleucina 18; TNF-α, Tumor necrosis factor-α.

The Table 6 shows the values of inflammatory cytokines in cases with estimated glomerular filtration rate (CKD-EPI) < 60 mL/min/1.73° m^2^. The HC group presented significantly elevated levels of the cytokines, except IL-18, compared to ContrHT (*P* < 0.05). On the other hand, the NT group had a very reduced number of patients with GFR < 60 mL/min/1.73° m^2^, fact that could harm the found results (only IL-6 was higher in the HC group when compared to NT group).

**TABLE 6:** Measurement of inflammatory cytokine levels in normotensive (NT), controlled hypertensive (ContrHT), and hypertensive crisis (HC) subjects. Group of patients with glomerular filtration rate (CKD-EPI) < 60 mL/min/1.73 m^2^.

	NT (a) (*N* = 3)		ContrHT (b) (*N* = 12)		HC (c) (*N* = 44)		Kruskal-Wallis test	*P*-value Multiple comparisons		
	Median	Range	Median	Range	Median	Range	*P*-value	a x b	a x c	b x c
IL-1β(pg/mL)	0.02	0.01; 0.20	0.05	0.00; 1.43	0.30	0.05; 6.82	**<0.001**	1.000	0.059	**0.002**
IL-6 (pg/mL)	1.22	0.37; 1.37	1.40	0.02; 22.66	12.42	1.40; 181.0	**<0.001**	1.000	**0.017**	**0.001**
IL-8 (pg/mL)	18.48	17.48; 20.24	11.36	4.85; 39.84	37.40	8.13; 1858.00	**<0.001**	1.000	0.505	**<0.001**
IL-10 (pg/mL)	4.07	1.49; 4.36	0.92	0.36; 6.65	7.81	0.96; 118.0	**<0.001**	1.000	0.432	**<0.001**
IL-18 (pg/mL)	13.50	7.20; 36.10	18.93	2.63; 135.94	23.97	1.13; 63.30	0.320	-	-	-
TNF-α(pg/mL)	21.27	9.19; 30.36	12.51	2.47; 24.15	25.08	2.69; 180.00	**0.004**	0.899	1.000	**0.003**

The bold values mean that the *P*-value is significant.

CKD-EPI, Chronic Kidney Disease Epidemiology Collaboration; IL-1 β, Interleukin 1β; IL-6, Interleukin 6; IL-8, Interleukin 8; IL-10: Interleukin 10, IL-18: Interleucina 18; TNF-α, Tumor necrosis factor-α.

The Table 7 presents the values of inflammatory cytokines in cases with GFR ≥ 60 mL/min/1.73° m^2^. All the cytokines were higher in the HC group compared to the control groups (NT and ContrHT) (*P* < 0.05).

**TABLE 7:** Measurement of inflammatory cytokine levels in normotensive (NT), controlled hypertensive (ContrHT), and hypertensive crisis (HC) subjects. Group of patients with glomerular filtration rate (CKD-EPI) ≥ 60 mL/min/1.73 m^2^.

	NT (a) (*N* = 64)		ContrHT (b) (*N* = 61)		HC (c) (*N* = 81)		Kruskal-Wallis test	*P*-value Multiple comparisons		
	Median	Range	Median	Range	Median	Range	*P*-value	a x b	a x c	b x c
IL-1β(pg/mL)	0.07	0.00; 5.54	0.05	0.00; 14.40	0.22	0.02; 81.35	**<0.001**	0.814	**<0.001**	**<0.001**
IL-6 (pg/mL)	0.64	0.00; 51.57	0.64	0.00; 20.33	3.87	0.27; 386.00	**<0.001**	1.000	**<0.001**	**<0.001**
IL-8 (pg/mL)	11.41	1.07; 63.73	8.78	2.72; 49.96	20.56	4.03; 5034.00	**<0.001**	1.000	**<0.001**	**<0.001**
IL-10 (pg/mL)	2.32	0.82; 37.91	1.19	0.07; 158.00	4.04	0.32; 335.00	**<0.001**	**0.021**	**0.001**	**<0.001**
IL-18 (pg/mL)	16.40	0.30; 93.70	9.98	1.17; 210.77	20.40	4.1; 129.50	**<0.001**	**0.009**	**0.006**	**<0.001**
TNF-α(pg/mL)	14.20	2.32; 32.64	12.27	4.45; 25.04	19.10	4.25; 264.00	**<0.001**	0.210	**<0.001**	**<0.001**

The bold values mean that the *P*-value is significant.

CKD-EPI, Chronic Kidney Disease Epidemiology Collaboration; IL-1 β, Interleukin 1β; IL-6, Interleukin 6; IL-8, Interleukin 8; IL-10, Interleukin 10; IL-18, Interleucina 18; TNF-α, Tumor necrosis factor-α.

The Table 8 shows the correlations between eGFR and inflammatory markers. There were negative correlations between eGFR and IL-1β (*r* = −0.20; *P* = 0.023), IL-6 (*r* = −0.34; *P* < 0.0001), IL-8 (*r* = −0.26; *P* = 0.003), IL-10 (*r* = −0.28; *P* = 0.001), and TNF-α (*r* = −0.36; *P* < 0.0001) in the HC group. The Table 9 demonstrates the correlation between age and inflammatory markers in HC group. A moderate positive correlation was found between age and IL-6 and a weak positive correlation between age and IL-10.

**TABLE 8:** Correlation between estimated glomerular filtration rate (eGFR) and inflammatory markers in the controlled hypertensive, and HC groups.

	eGFR (CKD-EPI)	*r*_s_	*P*-value	CI 95%
ContrT	IL-1β	0.06	0.598	−0.17;0.28
	IL-6	0.10	0.388	−0.13;0.32
	IL-8	−0.13	0.259	−0.35;0.10
	IL-10	−0.09	0.405	−0.32;0.13
	IL-18	0.09	0.421	−0.14;0.32
	TNF-α	−0.16	0.169	−0.38;0.07
HC	IL-1β	−0.20	**0.023**	−0.36; −0.02
	IL-6	−0.34	**<0.001**	−0.49; −0.17
	IL-8	−0.26	**0.003**	−0.41; −0.08
	IL-10	−0.28	**0.001**	−0.43; −0.10
	IL-18	0.03	0.718	−0.14;0.20
	TNF-α	−0.36	**<0.001**	−0.50; −0.19

Spearman’s correlation (r_s_); *P*-value < 0.05; The bold values mean that the *P*-value is significant.

CKD-EPI, Chronic Kidney Disease Epidemiology Collaboration; ContrHT, Controlled hypertensive; HC, hypertensive crisis; IL-1 β, Interleukin 1β; IL-6, Interleukin 6; IL-8, Interleukin 8; IL-10, Interleukin 10; IL-18, Interleukin 18; TNF-α, Tumor necrosis factor-α.

**TABLE 9:** Spearman’s correlation between age and inflammatory markers in the hypertensive crisis group.

	Age	*r*_s_	*P*-value	CI 95%
HC	IL-1β	0.039	0.661	−0.136; 0.211
	IL-6	0.250	**0.004**	0.077; 0.408
	IL-8	0.002	0.978	−0.172; 0.175
	IL-10	0.170	0.054	0.005; 0.335
	IL-18	0.015	0.871	−0.160; 0.189
	TNF-α	0.013	0.885	−0.161; 0.186

Spearman’s correlation (r_s_); *P*-value < 0.05; The bold values mean that the *P*-value is significant.

HC, hypertensive crisis; IL-1 β, Interleukin 1β; IL-6, Interleukin 6; IL-8, Interleukin 8; IL-10, Interleukin 10; IL-18, Interleukin 18; TNF-α, Tumor necrosis factor-α.

**FIGURE 2:**
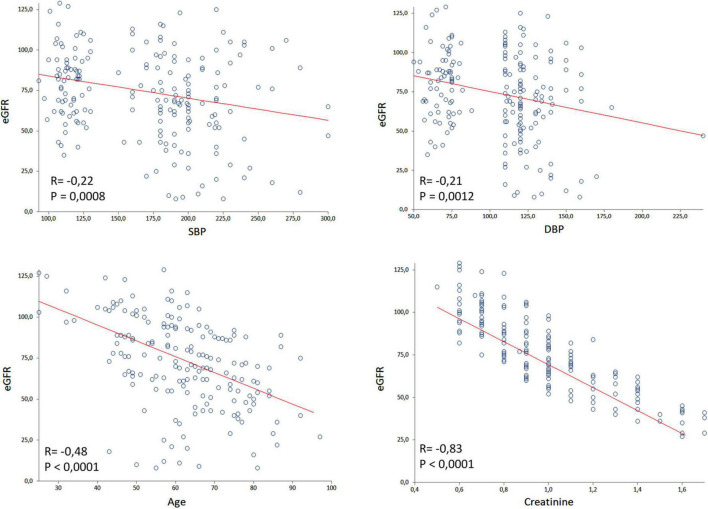
Spearman’s correlation of glomerular filtration rate (eGFR) with systolic blood pressure (SBP), diastolic blood pressure (DBP), age and creatinine considering controlled hypertensive, hypertensive urgency, and hypertensive emergency subjects. Outliers creatinine levels were excluded by the GraphPad Prism.

The Figure 2 shows the inverse correlation between eGFR and age (*r* = −0.54; *P* < 0.0001), SBP (*r* = −0.30; *P* < 0.0001), and DBP (*r* = −0.28; *P* < 0.0001). These correlations were made using the ContrHT, HUrg, and HEmerg groups. Outliers creatinine levels were excluded for this analysis.

## Discussion

This study shows elevated levels of proinflammatory cytokines associated to reduced renal function in individuals suffering from acute hypertensive events. Despite HC being an event common in the clinical emergency department (14–17), its pathophysiology continues unclear. Generally, this process is started by an imbalance between cardiac outcome and peripheral vascular resistance with participation of several mechanisms, including renal sodium handling, systemic vascular responses, cardiac output, and sympathetic outflow (4, 5). In addition, sudden increases in BP overlapping pre-existing hypertension appears to trigger deterioration of the kidney function (18).

In the present study, markers of kidney function (creatinine and eGFR) were altered in individuals suffering from HC. This group demonstrated upper creatinine levels and decrease in the eGFR compared to the control groups (NT and ContrHT), data confirmed by other studies investigating acute elevations of BP (18, 19). Hypertension and kidney function are deeply related and the association between them enhances cardiovascular risk; lower kidney function may trigger HC, and HC can acutely injury the kidneys. Individuals with HC have increased plasma aldosterone and renin, indicating neuro-hormonally mediated vascular lesion, and renal dysfunction (20). Thus, kidney injury may result from changes in renal vascular self-regulation, which is impaired in cases of acute BP elevation. Other mechanisms implicated in vascular damage and renal dysfunction include immunity-mediated and inflammatory fibrosis (5, 20).

In this work, it was not possible to discover whether the renal dysfunction was due to pre-existing chronic renal disease or acute renal failure, presenting as a manifestation of the HEmerg (21). Anyway, one third of hospitalized subjects with acute increases of BP course with > 25% reductions in renal function and represent a group with higher cardiovascular risk (9). So, kidney dysfunction is an independent predictor of mortality among individuals with HEmerg (22). Additionally, the risk of developing end-stage renal disease in patients with malignant hypertension, one presentation of HEmerg, is markedly elevated (23). Although the current study was not longitudinal, creatinine, and BP also correlated negatively to eGFR. Acute kidney disease caused by malignant hypertension may also reflect difficulty over the long term to regulate BP in hypertensive individuals, a fact observed in this study. Moreover, patients with HC were older than the individuals in the other groups as reported in other studies (14, 15). Thus, the growing number of older patients with HC shows that the incidence of cardiovascular disease increases almost linearly with increasing age (24). Complementing these data, the present study found a positive correlation between age and IL-6, a fact that may predispose to chronic inflammation and increased risk of morbidity and mortality (25). Hence, HC comprise a group of more severely ill subjects, with a higher prevalence of difficult-to-control hypertension and a greater propensity to develop cerebrovascular and renal complications.

In this research, all proinflammatory cytokines were increased in patients with acute elevations in BP (HUrg and HEmerg groups) compared to control subjects (NT and ContrHT groups). Only the IL-1β was significantly higher in the HEmerg group in comparison to the HUrg group. Effects associated with hypertension, such as increased vascular permeability, thrombogenesis and fibrosis, can be attributed to the chronic inflammatory process, which triggers endothelial dysfunction. Elevated serum level of proinflammatory cytokines in hypertensive patients has been associated with elevated BP values and/or TOD (26, 27). Thus, patients with chronic hypertension already present a latent inflammatory state, and possibly during an acute elevation of BP, occurs an exacerbation of this inflammatory process. In our study, it is possible that inflammatory cytokines were already close to maximum levels during a HC, and as observed, even in cases of HEmerg there was no difference in the cytokine levels in relation to cases of HUrg, despite the severity of clinical presentation.

IL-1β is an “early-response” cytokine which is produced by macrophages and monocytes, promotes the release of IL-6 as consequence of oxidative stress, a cardinal process in the pathogenesis of hypertension (26, 28, 29). In turn, IL-6 seems to be involved in the pathogenesis of hypertension through its effects on vascular inflammation and stiffness, and endothelial dysfunction. Moreover, it stimulates arterial wall collagen synthesis, inhibits the degradation of fibrinogen, and can promote a prothrombotic state. In addition, it may amplify the proinflammatory state by stimulating the production of acute phase proteins like C-reactive protein (CRP) (26). The IL-8 participates in the pathogenesis of atherosclerosis in hypertensive individuals and stimulates production of reactive oxygen species (30, 31). In turn, TNF-α can decrease the expression of endothelial nitric oxide (NO) synthase, resulting in reduction of the bioavailability of NO and leading to endothelial dysfunction, and consequently, elevation of BP (32). Unlike other cytokines, IL-10 is known to be the main anti-inflammatory cytokine. Its anti-inflammatory effects consist of inhibiting the activation of nuclear factor-kappa beta (NFkB) and suppressing the production of proinflammatory cytokines (IL-1β, IL-6, TNF-α, and adhesion molecules). Its increase helps to interrupt the inflammatory process triggered by a great inflammatory activity during the HC (26, 33, 34).

In this study, we observed greater inflammatory process in individuals with HC, which presented lower GFR compared to NT and ContrHT groups. However, there was no significant difference related to renal function (eGFR ≥ and < 60 ml/min) and cytokines into HC group. Some studies have shown that inflammation markers and oxidative stress are elevated in patients with kidney disease (11, 35). However, in our study this may not have been evident due to the reduced number of patients evaluated with eGFR < 60 mL/min/1.73° m^2^. Circulating monocytes and tissue macrophages (myeloid cell populations) from the innate immune system exacerbate both elevations in BP and TOD in hypertension thereby promoting endothelial dysfunction and provoking sodium retention in the kidney by disrupting renal blood flow (36–38). On the other hand, T-lymphocytes can directly regulate the BP inducing hypertension through vascular dysfunction (39, 40), while B-lymphocytes can alter BP indirectly by facilitating T-cell activation and consequent cytokine generation (adaptive immune system) (41–43). In turn, cytokines can modulate the salt-water balance by modifying sympathetic tone and renal nerve activity, by provoking endothelial dysfunction with secondary effects on renal blood flow, and/or by increasing sodium transport in the nephron (44), mechanisms involved in the pathophysiology of HC.

Similarly, all the cytokines were also higher in the HC group compared to control groups with GFR ≥ 60 mL/min/1.73° m^2^. In general, cytokines can regulate the synthesis of other pro-inflammatory molecules and, consequently, active several inflammatory pathways and generate vascular damage. Thus, IL-18 can regulate the synthesis of IL-1β and TNF-α, and interferon (IFN) G (45), in turn increasing chemokine receptors in mesangial renal cells (46). Furthermore, IL-8 increases the expression of intercellular adhesion molecule 1 (ICAM-1) (47) and promotes endothelial cell apoptosis (48). Interestingly, the elevated expression of ICAM-1 was observed in cases of the hypertensive emergencies (49).

A higher percentage of individuals with HC took beta-blockers compared to diuretics, which were prescribed with increased frequency in ContrHT group. This difference of antihypertensive strategies was also found in previous study of our group (50). This can be explained by the origin of the patients. The controlled hypertensive group is followed up in a university service specialized in hypertension (hypertension clinic). On the other hand, individuals with hypertensive crisis admitted to the Clinical Emergency Department of the university hospital are referred from other clinical care services in the Hospital area. Therefore, they are not followed by specialists in hypertension and, probably, the physicians of primary care are not aware about the guidelines and prescribe more beta-blockers than diuretics as first choice of treatment. Interestingly, other studies carried out in the emergency department also showed a higher percentage of patients using beta-blockers in relation to diuretics in patients suffering from CH (15, 51).

This study has both strengths and limitations. Its strengths include the contribution of clinically relevant and previously unavailable data on the association between renal function and the inflammatory process in patients suffering from HC. The numbers of individuals evaluated in the four groups of this study are higher than in previously published studies, which gives consistency to the results obtained. In addition, the evaluation of different inflammatory cytokines and the inclusion of two control groups (NT and ContrHT) provide results that are more robust. Nevertheless, some limitations should be considered. First, a cross-sectional study does not allow the identification of cause-and-effect relationships. Second, the sample size of the HUrg group was smaller than the other groups because this study was performed in a tertiary referral hospital with a high number of complex cases, which made it difficult to enroll patients with HUrg. Nevertheless, it was still possible to identify a significant difference in variables. Furthermore, the age group was not similar among the studied groups, but this did not interfere in the eGFR results between the NT e ContrHT groups. Finally, more specific markers of kidney function (cystatin C and neutrophil gelatinase associated lipocalin) were not used.

To the best of our knowledge, this is the first study performed in individuals with HC associating renal function with inflammatory cytokines. Elevation of proinflammatory and anti-inflammatory cytokines appears to participate in the pathophysiology of acutely elevated BP, independently of reduction grade of kidney function. Our data confirm that the mechanism associated to development of the HC is complex and not yet fully elucidated. Understanding acute BP elevation as an inflammatory-based pathology gives rise to new therapeutic targets. In the future, new studies should elucidate the clinical benefits resulting from the reduction of the inflammatory process.

## Data availability statement

The raw data supporting the conclusions of this article will be made available by the authors, without undue reservation.

## Ethics statement

The studies involving human participants were reviewed and approved by Research Ethics Committee of Medical School in Sao Jose Rio Preto (FAMERP). The patients/participants provided their written informed consent to participate in this study.

## Author contributions

JV-M designed the study. All authors contributed equally to the literature review, data interpretation, creation of figures, writing of the manuscript, read, and approved the final draft.
